# Crosstalk between microRNA and DNA Methylation Offers Potential Biomarkers and Targeted Therapies in ALK-Positive Lymphomas

**DOI:** 10.3390/cancers9080100

**Published:** 2017-08-03

**Authors:** Coralie Hoareau-Aveilla, Fabienne Meggetto

**Affiliations:** 1 Inserm, UMR1037 CRCT, F-31000 Toulouse, France; coralie.hoareau-aveilla@inserm.fr; 2 Université Toulouse III-Paul Sabatier, UMR1037 CRCT, F-31000 Toulouse, France; 3 CNRS, ERL5294 CRCT, F-31000 Toulouse, France; 4 Laboratoire d’Excellence Toulouse Cancer-TOUCAN, F-31024 Toulouse, France

**Keywords:** epigenetics, DNA methylation, microRNA (miRNA), ALK, lymphomas, biomarkers, novel treatments

## Abstract

The discovery of microRNA (miRNA) has provided new and powerful tools for studying the mechanism, diagnosis and treatment of human cancers. The down-regulation of tumor suppressive miRNA by hypermethylation of CpG island (*CpG* is shorthand for *5′-C-phosphate-G-3′*, that is, cytosine and guanine separated by only one phosphate) is emerging as a common hallmark of cancer and appears to be involved in drug resistance. This review discusses the role of miRNA and DNA methylation in drug resistance mechanisms and highlights their potential as anti-cancer therapies in Anaplastic Lymphoma Kinase (ALK)-positive lymphomas. These are a sub-type of non-Hodgkin’s lymphomas that predominantly affect children and young adults and are characterized by the expression of the nucleophosmin (NPM)/ALK chimeric oncoprotein. Dysregulation of miRNA expression and regulation has been shown to affect several signaling pathways in ALK carcinogenesis and control tumor growth, both in cell lines and mouse models. These data suggest that the modulation of DNA methylation and/or the expression of these miRNA could serve as new biomarkers and have potential therapeutic applications for ALK-positive malignancies.

## 1. Introduction

Cancer initiation and progression is controlled by both genetic and epigenetic events. Epigenetic alterations are reversible and heritable modifications that influence gene expression and phenotype without changing the DNA sequence. Recent advances in epigenetics have not only offered a deeper understanding of the underlying mechanisms of carcinogenesis and cancer progression, but have also allowed the identification of clinically relevant putative biomarkers for early detection, disease monitoring, prognosis and risk assessment in patients [1,2].

Cancer epigenetics have shown extensive reprogramming of two interconnected epigenetic mechanisms, DNA methylation, and histone modifications. In addition, non-coding RNA, specifically microRNA (miRNA) expression is intimately involved in the repressive chromatin state [1]. Thus, enzymes involved in epigenetic modifications, such as DNA methyltransferases (DNMTs) or histone deacetylases (HDACs) and miRNA, have emerged and are considered as potential targets for cancer prevention and therapy [2].

Peripheral T-cell lymphomas (PTCLs) are a heterogeneous group of nodal and extranodal tumors that account for 10 to 15% of all non-Hodgkin lymphomas (NHLs). The three most common subtypes of PTCL, namely PTCL not otherwise specified, angioimmunoblastic T-cell lymphoma and anaplastic large-cell lymphoma (ALCL), represent about 70% of PTCL cases in Europe and the United States of America. ALCL is a biologically and clinically heterogeneous subtype of T-cell lymphoma characterized by large lymphoid cells expressing the Ki-1 (CD30) molecule [3,4]. The World Health Organization (WHO) has classified the ALCL into three major entities, primary systemic (in lymph nodes or organs throughout the body at presentation) ALK(+) (Anaplastic Lymphoma Kinase-positive) ALCL, primary systemic ALK(−) ALCL, and primary cutaneous (skin-only involvement without systemic dissemination at presentation). Primary cutaneous ALCL are always ALK(−) [5]. Of note, ALK(−) ALCL associated with breast implants (i-ALCL) has been recently recognized as a distinct entity [6,7].

Primary systemic ALK(+) ALCL account for approximately 3% of adult NHL cases and 10 to 20% of all childhood NHL cases [8]. This subtype is often (80%) characterized by the t(2;5)(p23;q35) chromosomal translocation, which leads to the expression of an 80 kDa chimeric NPM/ALK tyrosine kinase consisting of the N-terminal oligomerization motif of nucleophosmin (NPM) fused to the cytoplasmic kinase domain of ALK, a receptor tyrosine kinase (RTK) [8,9]. Native full-length ALK is a dependence receptor mainly expressed in discrete regions of the developing central and peripheral nervous system, but not in normal lymphoid cells [10]. ALK belongs to the insulin receptor family and is related to the leucocyte tyrosine kinase receptor (LTK) [11]. NPM is a ubiquitously expressed nucleolar protein that is involved in ribosomal components, shuttling between the cytoplasm and nucleus [8].

In ALCL, oncogenic NPM/ALK signaling is mediated by different pathways, including PI3K/Akt, MAPK (Mitogen-activated protein kinases), pp60c-src tyrosine kinase and STAT3 (signal transducer and activator of transcription 3) transcription factor. These NPM-ALK-activated signal pathways control key cellular processes such as proliferation and survival and promote lymphomagenesis in NPM/ALK(+) ALCL [8,12,13]. Polychemotherapy regimens containing doxorubicin, such as CHOP (cyclophosphamide, doxorubicin, vincristine, prednisone) or CHOEP (CHOP with etoposide), are standard first-line treatments for NPM/ALK(+) ALCL [14,15]. While these regimens achieve high remission rates, relapse and resistance occur in 20 to 40% of patients with invariably poor prognosis [8]. Clinical studies have supported ALK as an extremely attractive therapeutic target for NPM-ALK(+) ALCL, and several ALK inhibitors are actually at various stages of clinical development or have received FDA (US Food and Drug Administration) approval. Among them, the first-generation ALK inhibitor crizotinib was approved by the US FDA in 2011 for the treatment of ALK(+) non-small-cell lung cancer [16]. Crizotinib has so far proven to be efficacious in the treatment of NPM-ALK(+) ALCL in pediatric and young adult patients [8]. Nonetheless, the emergence of resistance is universal [17,18]. Therefore, understanding the cellular and molecular mechanisms leading to the development of NPM-ALK(+) ALCL is mandatory to discover potential biomarkers for early detection and consequently novel therapeutic targets to improve the clinical outcome of patients with ALK(+) diseases. The development of novel targeting strategies and new generation ALK inhibitors are thus necessary to counteract the emergence of resistance with ALK inhibitors [19,20].

Over the last decade, many studies have provided evidence for the involvement of DNA methylation and miRNA in ALK tumorigenesis. In addition, DNA methylation at certain genomic loci and miRNA expression patterns have been established as potential markers for the sub-classification, diagnosis, prognosis and identification of therapeutic targets in ALK(+) ALCL. Recent studies have highlighted the role of the specific miRNA methylation in oncogenic ALK signaling in ALK(+) ALCL [21], and even ALK itself has been proposed to be regulated by the miR-96 microRNA [22]. Thus, miRNA do not only influence the cellular phenotype and pathogenesis of ALK(+) ALCL but they are also emerging as tissue-specific biomarkers with potential clinical applications for both identifying cancer subtypes and developing new therapies [17]. In this paper, we focus on the interplay between DNA methylation and miRNA dysregulation and their potential benefits for certain clinical applications in NPM/ALK(+) ALCL.

## 2. microRNA in ALCL

The biogenesis of miRNA involves a complex process with multiple steps. miRNA genes are initially transcribed by RNA polymerase II to form primary-miRNA with hairpin structures. The DROSHA-DGCR8 enzyme complex then cleaves these primary-miRNA into precursor-miRNA, which are subsequently transported to the cytoplasm by Exportin-5 (XPO5) and cleaved by DICER to yield miRNA duplexes. One strand is then chosen to function as mature miRNA and is loaded into the RNA-induced silencing complex (RISC), whereas the partner miRNA* (the “*” notation indicates the passenger strand of the duplex) is degraded. Mature miRNA (miRNA) are 20–25 nucleotide-long non-coding RNA that induce the translational repression and/or degradation of target mRNA, thereby acting as negative regulators of gene expression [23,24]. MiRNA bind to the 3′ untranslated region of specific mRNA sequences. This interaction can lead to either targeted mRNA cleavage of targeted mRNA or repression of mRNA translation, in both cases resulting in reduced levels of the encoded protein. It has been predicted that miRNA account for 1–5% of the human genome and regulate at least 30% of protein-coding genes [25]. To date, 1881 distinct miRNA have been identified within the human genome (http://microrna.sanger.ac.uk). miRNA are known to be involved in many important biological processes, including different stages of hematopoietic development, regulation of cell differentiation, and apoptosis. In some tumors, including tumors of the hematopoietic and lymphoid tissues, altered miRNA expression is commonly observed, implying that miRNA may be involved in the hematological development of leukemia and lymphomas [24]. The role of miRNA in the process of malignant transformation has been well characterized. They can act as oncogenes and tumor suppressors depending on the cellular functions of their targeted gene [23]. In contrast to mRNA, miRNA are abundant not only in tissues but also in body fluids such as blood or urine. This easy accessibility through non-invasive liquid biopsies makes miRNA promising biomarkers in cancer screening [26]. Thus, expression profiling of miRNA is proving to be clinically relevant in cancer diagnosis, progression and outcome.

### 2.1. microRNA as Molecular Signatures of ALK-Positive ALCL

Based on the expression of miRNA, several studies have identified different gene signatures distinguishing ALK(+) from ALK(−) ALCL [27,28,29,30,31,32,33,34,35]. This discrepancy could reflect the variability of (i) ALCL models used in these studies—ALK(+) cell lines: SU-DHL-1, KARPAS-299, SR-786, SUP-M2/TS, JB-6, L82, KiJK, DEL, Pio and COST; ALK(−) cell lines: FE-PD, Mac1 and Mac2A [36]; Formalin-Fixed/Paraffin-Embedded (FFPE) tumor samples from both ALK(+) and ALK(−) ALCL and NPM/ALK transgenic mice—and (ii) methods employed to detect miRNA (microarrays, next generation sequencing or real time RT-PCR analyses). Only a limited number of miRNA seems to discriminate ALK(+) from ALK(−) ALCLs. Compared to ALK(−) ALCLs, miR-203, miR-135b, miR-886-5p/3p, miR-20b, miR-106a and miR-183 were significantly upregulated in ALK(+) ALCLs while others (miR-155, miR-181a, miR-210, miR-29a/b, miR-342-5p/3p, miR-369-3p miR-374a/b, miR-423-5p, miR-625, miR-205, miR-146a and miR-26a) were down-regulated (Table 1).

Furthermore, two studies have sought to determine an ALK-associated miRNA signature. The first one used a knockdown experiment and identified 32 miRNA that are differentially regulated in the SUP-M2/TS ALK(+) cell line (23 upregulated and nine downregulated) [29]. Several of the upregulated miRNA, such as the miR-17~92 cluster, are known to promote cell proliferation. In the second study, in addition to the knockdown of ALK, the authors investigated the implication of STAT3 in the ALK-dependent miRNA signature [34]. Using inducible sh-RNA targeting either ALK or STAT3 in the SUP-M2/TS cell line, this later study showed that a significant number of miRNA are co-regulated by ALK and STAT3 [30]. These results point out STAT3 as an essential effector in miRNA regulation by ALK. Surprisingly, only few miRNA identified as part of the signature distinguishing ALK(+) ALCL from T-cells or ALK(−) ALCL were affected by ALK knockdown: miR-20b, miR-106a, miR-886-5p and miR-181a. However, it is important to note that this study was conducted on a single cell line (SUP-M2/TS) and, as a consequence, could reflect the particularity of this cell line and by extension the uniqueness of the patient that the cell line was derived from. As an illustration of this concept, Steinhilber and colleagues showed only a partial overlap of dysregulated miRNA when 3 ALK(+) ALCL cell lines (SU-DH-L-1, KARPAS-299 and KiJK) were compared to T-cells or ALK(−) ALCL (Table 2 and Table 3).

Nevertheless, high-throughput studies of miRNA in ALCL have begun to unravel the signaling pathways involved in the regulation and effects of various miRNA in the development of this cancer.

### 2.2. Tumor Promoter microRNA in ALK-Positive ALCL

#### 2.2.1. The miR-17~92 Cluster

The miR-17~92 cluster families (miR-17~92 cluster itself and its paralog clusters miR-106a~363 and miR-106b~25) are part of the miRNA signature defining ALK(+) ALCL. First identified as being overexpressed in ALK(+) ALCL primary tumors and cell lines compared to T-cells and ALK(−) ALCL [33], these miRNA were then studied in vitro for their role in the growth of ALK(+) ALCL cell lines [34]. The main findings of this study was that the forced expression of the miR-17~92 cluster could partially rescue STAT3 knockdown by sustaining the proliferation and survival of NPM/ALK(+) cells both in vitro and in a xenograft mouse model. It also induced the downregulation of the pro-apoptotic protein BIM, suggesting that the miR-17~92 cluster might mediate resistance to STAT3 knockdown by targeting BIM [34]. Furthermore, pharmacological TNF inhibition of STAT3, using the STAT3 inhibitor Stattic, decreased miR-17~92 cluster expression in all of the ALK(+) ALCL cell lines tested (SUP-M2/TS, JB-6, L82, and KARPAS-299). The same results in SUP-M2/TS cells were also reported by Liu and collegues [29] (Table 2 and Table 3).

#### 2.2.2. miR-135b

When analyzing the differences of miRNA regulation in ALK(+) versus ALK(−) cells following inactivation of STAT3 by shRNA-mediated ALK or STAT3 knockdown, miR-135b expression was most prominently altered, with a significant upregulation in ALK(+) ALCL cell lines and human primary ALK(+) ALCL samples compared to ALK(−) ALCL cells [30,43]. MiR-135b targets the FOXO1 transcription factor in ALK(+) ALCL cell lines, which is critical as FOXO1 can promote the expression of the cell cycle inhibitors p21 and p27. Moreover, miR-135b suppressed the T-helper (Th) 2 master regulators STAT6 and GATA3, and inhibition of miR-135b suppressed the production of a Th17 pro-inflammatory cytokine, IL-17, by NPM/ALK cells. This indicates that miR-135b-mediated Th2 suppression may lead to the ALCL immunophenotype overlapping with Th17 cells [30]. In accordance with the pro-angiogenic function of IL-17, miR-135b inhibition also reduced tumor angiogenesis and growth in vivo (Table 3).

Together, these data suggest that miRNA could sustain the oncogenic properties of STAT3 in ALCL and that STAT3 inhibition might represent an alternative avenue for interfering with ALK signaling in ALCL. Thus, the combination of a small molecule-based inhibitor of ALK with either a STAT3 inhibitor or an miRNA inhibitor may be a useful strategy to prevent chemoresistance in patients with ALK(+) ALCL and other ALK(+) malignancies.

#### 2.2.3. miR-155

miR-155, showed a significantly higher expression in ALK(−) ALCL compared to ALK(+) ALCL [31,33]. An inverse correlation between miR-155 promoter methylation and miR-155 expression was reported in ALK(+) ALCL, but no direct effect of the ALK kinase on methylation of the *MIR155* gene was observed [32]. Overexpression of miR-155 in ALCL ALK(+) cell lines was shown to reduce levels of the transcription factor C/EBPβ (CCAAT/enhancer binding protein beta) and the suppressor of cytokine signaling-1 (SOCS1) [32]. In murine engraftment models of ALK(−) ALCL, anti-miR-155 molecules reduced tumor growth, raised tumoral levels of cleaved caspase-3 and increased SOCS1 expression, leading to the suppression of STAT3 signaling. Moreover, miR-155 was found to induce interleukin-22 (IL-22) expression and suppress the C/EBPβ target interleukin-8 (IL-8), two pro-inflammatory cytokines that are also endogenously produced by ALK(+) ALCL cells [44,45] (Table 2 and Table 3). These data suggest that miR-155 can act as a driver of tumorigenesis in ALCL ALK(−), making it the first molecular therapeutic target for ALK(−) ALCL [19,32].

#### 2.2.4. miR-203

Two independent studies found miR-203 to be significantly upregulated in both ALK(+) ALCL cell lines and primary tumors but not expressed in normal T-cells, the ALK(−) ALCL cell line Mac-1 and ALK(−) primary cases [27,35]. Although its function remains elusive, miR-203 seems to play a role in the immune response through the regulation of SOCS-3 [35]. Furthermore, miR-203 was identified by Steinhilber and colleagues as part of a signature of three miRNA (miR-181a, miR-146b-5p and miR-203) significantly regulated by the C/EBPβ transcription factor, which is specifically overexpressed in ALK(+) ALCL cell lines and shown to promote tumoral cell proliferation and survival (Table 2 and Table 3).

### 2.3. MicroRNA Suppressors of ALCL Development

#### 2.3.1. miR-101, miR-29a and miR-150: Suppressors of Cell Proliferation and Survival

miR-101 was found to be down-regulated in ALK(+) and (−) human ALCL and CD4/NPM/ALK transgenic mouse models, but its forced expression increased the number of cells arrested in G1 phase of the cell cycle only in ALK(+) and not in ALK(−) ALCL cell lines The serine/threonine kinase mTOR was shown to be targeted by miR-101, and its inhibition led to reduced tumor growth in engrafted ALCL mouse models, suggesting that mTOR inhibitors may offer a viable therapeutic strategy [31]. This attenuated cell proliferation was also likely to be a result of downregulation of miR-101 targets, such as the pro-survival protein Mcl-1 [31]. Our laboratory showed for the first time that ALK(+) ALCL cell lines (SU-DHL-1, KARPAS-299, Pio and COST) and biopsy specimens express low levels of miR-29a [42]. We found that the repression of miR-29a is dependent on NPM/ALK and STAT3, as demonstrated by siRNA-mediated ALK or STAT3 knockdown (further described in paragraph 3) [42]. Enforced miR-29a expression was shown to modulate apoptosis through repression of the pro-apoptotic factor MCL-1 in ALCL cell lines in a xenograft model, with a concomitant reduction in tumor growth. Decreased MCL1 expression also correlated with an increased sensitivity of cells to doxorubicin [42].

Levels of another miRNA, miR-150, were also shown to be reduced in ALK(+) ALCL cell lines and biopsy specimens as a consequence of the activity of the ALK kinase (further described in paragraph three) [37]. We could show that miR-150 is a tumor suppressor miRNA in ALK(+) ALCL cells. Indeed, miR-150 overexpression led to a drastic inhibition of cell growth and tumor formation through repression of its downstream target MYB, a master regulator of proliferation in ALK(+) ALCL cells [37] (Table 3).

#### 2.3.2. miR-181a and miR-16 as Regulators of Tumor Microenvironment

miR-181a regulates T-cell differentiation and modulates TCR (T Cell Receptor) signaling strength. It was shown to be significantly downregulated in a large proportion of ALK(+) ALCL tumors. In addition, this miRNA is one of the three miRNA (miR-181a, miR-146b-5p and miR-203) regulated by the C/EBPβ transcription factor. Thus, the decreased expression of miR-181a and the increased expression of miR-203 in ALK(+) ALCL might provide a mechanism by which these tumors escape immune surveillance. We have also demonstrated that NPM/ALK modulates the tumor microenvironment, including the stimulation of angiogenesis [38,45]. Indeed, using our conditional NPM/ALK lymphoma transgenic mouse model [46,47], we observed that ALK works alongside HIF1α to boost VEGF (Vascular endothelial growth factor) expression by down-regulating miR-16, an inhibitor of VEGF mRNA [38]. Moreover, injection of miR-16 into the tumors of nude mice was found to decrease tumor growth in vivo [38]. These studies demonstrate how antiangiogenic and vascular normalization therapies may become attractive therapeutic targets in ALK(+) ALCL [48] (Table 3).

#### 2.3.3. MiR-21, miR-26a and miR-219

miR-21, miR-26a and miR-219 are also known to be repressed by an NPM/ALK/STAT3-dependent mechanism, and their targeting could represent another way to improve therapeutic approaches. In SU-DHL-1cells, NPM/ALK induces the expression of ICOS, a T-cell growth-promoting costimulatory receptor that amplifies the signal generated by the antigen-specific TCR, and NPM/ALK-mediated activation of STAT3 suppresses the expression of the ICOS inhibitor miR-219 [40]. The downregulation of NPM/ALK or STAT3 by siRNA was also associated with a significant increase in miR-26a levels and a marked decrease in levels of the iNOS (Nitric oxide synthase) protein, which promotes lymphocyte survival and protects them from apoptosis [41]. Restoration of miR-26a in ALK(+) cells was associated with a marked decrease in iNOS protein expression, and this was associated with a reduction in nitric oxide (NO) release and a decrease in viability, adhesion to endothelial cells and migration. Moreover, negligible levels of miR-26a were detected in ALK(+) ALCL cell lines (KARPAS-299 and DEL) and tumor samples. On the contrary the expression of the iNOS protein was found to be pronounced in these samples (Table 3).

## 3. DNA Methylation Deregulation in ALK-Positive ALCL

Epigenetic alterations are frequently found in diverse malignant diseases, and epigenetic gene silencing plays a key role in inhibiting tumor suppressor gene expression in cancer cells [49]. Gene silencing usually takes place at promoter regions and is executed by two principal mechanisms: methylation of the DNA within the CpG-enriched sequences, and histone modification. In the mammalian genome, the covalent addition of a methyl group takes place at cytosine residues that are located next to a guanine residue (CpG dinucleotide). DNA methylation of the promoter region inhibits the transcription of their genes, including miRNA genes [50]. In cancer, the methylation of tumor suppressor gene regulatory regions often leads to their downregulation, potentially playing a pathogenic role in carcinogenesis and the resistance to apoptosis-inducing drugs [51]. Both losses and gains in DNA methylation are often found concurrently in cancer. Indeed, the DNA methylation profile of tumors is frequently characterized by global hypomethylation and simultaneous hypermethylation of CpG islands, specific genomic regions containing a high frequency of CpG sites. A loss of methylation affects repetitive genomic elements, intergenic regions and gene bodies, whereas methylation gains mostly occur in the promoter, the 5′ untranslated region (5′UTR) or the first exons/introns of a gene, explaining why gene methylation and transcription are usually inversely correlated. Nearly 70% of annotated gene promoters in the human genome contain a high CpG content.

DNA methylation is directly mediated by three members of the DNMT family, DNMT1, DNMT3A, and DNMT3B, which carry out and maintain the methylation of CpG dinucleotides [52,53]. DNA methylation can be reversed by treatment with DNMT inhibitors such as 5-azacytidine (5-aza-CR, Vidaza^®^, Celgene Corp., Summit, NJ, USA) or the more stable 5-aza-2′-deoxycytidine (5-aza-CdR, decitabine, Dacogen^®^, SuperGen, Inc., Dublin, CA, USA), which become incorporated into the DNA of actively proliferating cells. Upon incorporation, they form covalent complexes with DNA methyltransferases and thus trap the enzymes at DNA sites. At low doses they inhibit the propagation of DNA methylation during each round of replication, whereas at high doses cytotoxic side effects can occur [54]. In NPM/ALK(+) cell lines and/or malignant lymph node biopsies from patients, promoter methylation has been shown to silence genes important for cell proliferation and survival, such as the cell cycle inhibitor p16INK4a, the cytokine TNFα which can trigger apoptosis, and both nuclear factors of activated T-cells 1 (NFATC1) and BIM, two proteins which transduce pro-apoptotic signals (Table 3) [39,55,56,57]. NPM/ALK was also shown to induce BIM methylation through the recruitment of a co-repressor complex including HDAC1/2 in association with the transcriptional regulatory protein SIN3a, however NPM/ALK was dispensable for maintaining BIM epigenetic silencing [57].

In other mechanistic models, NPM/ALK has been implicated in the upregulation and recruitment of DNMT1 to the promoters of genes such as SHP1, STAT5A and ILR2γ, via its activation of the transcription factor STAT3 (Table 3) [39,58,59]. STAT3 was also shown to form a complex with HDAC-1 and DNMT1 to bind and repress SHP1 transcription. The restoration of SHP1 expression by 5-aza-2′-CdR was associated with the downregulation of STAT3 signaling [58,60]. The protein products of the *SHP1*, *STAT5A* and *ILR2γ* genes act as tumor suppressors by interfering with the expression of NPM/ALK [39,58,59]. The NPM/ALK-STAT3 axis also promotes methylation-induced silencing of several components of T-cell receptor (TCR) signaling and T-cell identity such as ZAP70, CD3 and SLP76 (Table 3) [61]. More recently, a genome-wide DNA methylation analysis reported the hypermethylation of the gene coding for the TCR co-stimulatory protein CD28 and the immune checkpoint receptor CTLA-4 in primary NPM/ALK(+) ALCL (Table 3) [62]. Thus, accumulating data shows that the DNA methylation and silencing of tumor suppressor genes plays a key role in NPM/ALK-induced malignant transformation. Accordingly, human ALCL cell lines and primary tumors were found to have high DNMT1 expression, and gene silencing in NPM/ALK(+) cell lines was shown to be reverted by demethylating drugs, even at low doses [63].

## 4. Crosstalk between microRNA and DNA Methylation Reveals Potential Targeted Therapies and Biomarkers for ALK-Positive Cancers

Differential expression of miRNA in cancer cells can be caused by several mechanisms, most notably genetic instability (amplification, deletion or translocation). Approximately 50–70% of miRNA genes are located at fragile genomic sites that are frequently affected by copy number alterations. In addition, a number of transcription factors regulate miRNA transcription, and their dysregulation in cancer cells in turn affects the expression of miRNA [65]. Following activation by NPM/ALK, STAT3 has a profound inhibitory effect on miRNA gene expression by recruiting the epigenetic gene silencing complex containing DNMT to gene promoters [21,37,42]. In ALK(+) cells, STAT3 activation enhances the general activity of the gene silencing machinery by promoting the expression of DNMT1, the key component of this machinery, by both directly activating the *DNMT1* gene and suppressing the expression of the DNMT1 mRNA inhibitor miR-21 [39].

Our laboratory showed for the first time that ALK(+) ALCL cell lines and primary tissues express low levels of miR-29a likely mediated by the hypermethylation of the *MIR29A* promoter dependent on NPM/ALK activity and STAT3. This repressive methylation is catalyzed by DNMT1 and DNMT3b and can be partially reversed by treatment with 5aza-CdR and, albeit to a lesser extent, after NPM/ALK knockdown by siRNA [42]. As mentioned in Section 2.3.1 the main consequence of miR29a loss in ALK(+) ALCL cells is an increase of cell survival via the high expression of the anti-apoptotic factor MCL-1, a bona fide miR-29a target. Levels of another tumor suppressor miRNA, miR-150 (mentioned in Section 2.3.1), were also reduced in the NPM/ALK(+) ALCL cell lines and biopsy specimens as a consequence of DNA hypermethylation, with DNA hypermethylation-mediated miR-150 repression also shown to require ALK-dependent pathways [37]. Moreover, epigenetic silencing of miR-150 was due to the activation of STAT3 which regulates DNMT1 expression. Accordingly, miR-150 repression was turned off following treatment with the DNMT inhibitor, decitabine, and in murine NPM/ALK(+) xenograft models miR-150 upregulation was found to induce anti-neoplastic activity [37]. Furthermore, both the treatment of crizotinib-resistant NPM/ALK(+) KARPAS-299-CR06 cells with decitabine and ectopic miR-150 expression reduced viability and growth [37].

In conclusion, the aberrant DNA methylation of specific miRNA genes offers potentially useful biomarkers for detecting cancer and predicting its outcome. This knowledge is essential for selecting vulnerable targets and addressing innovative therapies such as hypomethylated agents. Indeed, our results suggest that a hypomethylating therapeutic strategy, either alone or in combination with other agents, may benefit ALK(+) patients that harbor tumors resistant to crizotinib and other anti-ALK tyrosine kinase inhibitors.

## 5. Conclusions

In this review we have discussed recent work on the DNA methylation of coding and non-coding genes with a particular interest in the aberrant DNA methylation of miRNA. The last two have been found to be associated with carcinogens and therapeutically-targeted molecules in NPM/ALK(+) ALCL. Because miRNA are far less degraded in FFPE samples than mRNA and are present not only in cancer tissues but also in bodily fluids, miRNA analyses offer more diagnostic potential for cancer research. Furthermore, the activation of tumor-suppressive miRNA and the inhibition of oncogenic miRNA may have the potential to provide a fundamentally new approach for the development of therapeutics for many cancers, including ALK(+) ALCL. Further research on the use of methylated miRNA genes as diagnostic markers and the feasibility of conducting hypomethylated treatments in clinical practice is warranted. In addition, we have also introduced the idea of methylated-miRNA as modulators of the tumor immune response. Epigenetic therapy has recently been reinvigorated due to its reported ability to induce viral mimicry in its exploitation as a priming agent for targeted immune checkpoint modulation [66]. In this connection, the repression of the immune checkpoint inhibitor CTLA-4 by DNA methylation in ALCL can provide appropriate justification for the epigenetic therapy with the objective of re-sensitizing lymphoma cells to checkpoint regulation [62]. Given the all but inevitable ALK inhibitor resistance, it is more than likely that drug combination therapies targeting not only ALK but also DNA methylation will be required in order to achieve durable complete remission or cure in the majority of patients with ALK(+) ALCL and other ALK-driven malignancies.

## Figures and Tables

**Table 1:** Distinct microRNA (miRNA) signatures differentiating in Anaplastic Lymphoma Kinase (ALK), positive from ALK(−) Anaplastic Large Cell Lymphomas (ALCL).

Sample	Normal Counterpart	Technique Used	Signature of microRNA		Ref.
Primary tumors: 21 ALK(+) ALCL and 23 ALK(−) ALCL	T-cells from healthy donors	TaqMan Array Human MicroRNA Card A v.2.0 (Life Technologies, Carlsbad, CA USA)	up-regulated in ALK(+) versus ALK(−) ALCL: let-7f, miR-138, miR148a, miR-203, miR-224, miR-340, miR-372, miR-376c, miR-505, miR598,	down-regulated in ALK(+) versus ALK(−) ALCL: miR-105, miR-124, miR-125a-5p, miR-129-5p, miR-132, miR-136, miR-145, miR-147, miR-147b, miR-149, miR-155, miR-181a, miR-185, miR-181c, miR-188-3p, miR-197, miR-199a-3p, miR-202, miR-208, miR-208b, miR-210, miR-214, miR-216a, miR-220b, miR-220c, miR-223, miR-296-5p, miR-298, miR-299-3p, miR-29a, miR-29b, miR-320, miR-324-3p, miR-325, miR-326, miR-328, miR-342-5p, miR-34a, miR-369-3p, miR-374a, miR-374b, miR-376b, miR-377, miR-380, miR-381, miR-384, miR-412, miR-423-5p, miR-448, miR-450-3p, miR-453, miR454, miR-455-3p, miR-485-5p, miR-487a, miR-491-3p, miR-492, miR-493-5p, miR-494, miR-499-3p, miR-499-5p, miR-501-3p, miR-507, miR-508-5p, miR-509-5p, miR-510, miR-511, miR-512-5p, miR-513-5p, miR-515-3p, miR-516a-5p, miR-516b, miR-517b, miR-518a-5p, miR-518c, miR-518d-3p, miR-518f, miR-519e, miR-520a-3p, miR-520b, miR-520d-5p, miR-520e, miR-523, miR-524-5p, miR-525-5p, miR-526b, miR-541, miR-544, miR-545, miR-548a-3p, miR-548a-5p, miR-548b-3p, miR-548b-5p, miR-548c-5p, miR-548d-5p, miR-556-3p, miR-556-5p, miR-561, miR-570, miR-576-3p, miR-582-5p, miR-590-5p, miR-597, miR-615-3p, miR-615-p, miR-618, miR-624, miR-625, miR-636, miR-652, miR-654, miR-674, miR-871, miR-872, miR-874, miR-875, miR-876, miR-876-3p, miR-885-3p, miR-885-5p, miR-887, miR-890, miR-891b, miR-892a, miR-92a	[27]
Primary tumors: 17 ALK(+) ALCL and 18 ALK(−) ALCL	Lymph nodes from healthy donors	Human miRNA Microarray version 3 (Agilent Technologies, Santa Clara, CA) chips	up-regulated in ALK(+) ALCL: miR-486-5p, miR-500-3p and miR-629	up-regulated in ALK(−) ALCL: miR-29a, miR-29b-1-5p, miR-155, miR-720	[31]
Primary tumors: 33 ALK(+) ALCL and 25 ALK(−) ALCL	T-cells from healthy donors	TaqMan Array Human MicoRNA A Card, V2.0; ABI	up-regulated in ALK(+) ALCL versus T-cells: miR-10b, miR-107, miR-124, miR-134, miR-137, miR-204, miR-323-3p, miR-337,5p, miR-376a, miR-379, miR-411, miR-485-3p, miR-495, miR-503, miR-512-3p, miR-517a, miR-517c, miR-518e, miR-518f, miR-519a, miR-539	down-regulated in ALK(+) ALCL versus T-cells: miR-98, miR-184, miR-200a, miR-205, miR-342-5p, miR-375, miR-486-5p, miR-486-3p, miR-501-5p	[29]
			up-regulated in ALK(+) versus ALK(−) ALCL: miR-135b, miR-512-3p, miR-708, miR-886-5p, miR-886-3p	down-regulated in ALK(+) versus ALK(−) ALCL: miR-146a and miR-155	
Primary tumors: 8 ALK(+) ALCL and 5 ALK(−) ALCL	Lymph node from healthy donors	LNA-modified miRCURY LNA miRNA Array ready-to-spot probe set no. 208010-A (Exiqon)	up-regulated in ALK(+) ALCL versus T-cells: miR-886-3p, miR-20b, miR-17, miR-106a, miR- 20a	down-regulated in ALK(+) ALCL versus T-cells: miR-451, miR-145, miR-146a, miR-142-3p, miR-29c, miR-29a, miR-29b, miR-30a, miR-342-3p, miR-26a, miR-142-5p, miR-101, miR-150, miR-155	[32]
ALK(+) cell lines (KARPAS-299, SU-DHL-1 and SR786) and ALK(−) ALCL cell lines (FEPD and Mac2a)	T-cells from healthy donors	LNA-modified miRCURY LNA miRNA Array ready-to-spot probe set no. 208010-A (Exiqon)	up-regulated in ALK(+) ALCL versus T-cells: miR-886-3p, miR-886-5p, miR-432*, miR-363*, miR-18a, miR-183, miR-302c*, miR-20b, miR-525-5p, miR-20a, miR-106a, miR-17-1	down-regulated in ALK(+) ALCL versus T-cells: miR-146a, hsa-miR-142-3p, miR-640, miR-518e*/519a*/519b-5p/, miR-423-3p, miR-423-5p, miR-125a-5p, miR-30c, miR-206, miR-215, miR-452, miR-29b, miR-146b-5p, miR-374b, miR-29a, miR-142-5p, miR-29c, let-7i, miR-30a, miR-374a, miR-101, miR-140-5p, let-7g, miR-22, miR-26a, miR-125b, miR-342-3p, miR-369-3p, miR-150	
			up-regulated in ALK(+) ALCL versus ALK(−) ALCL: miR-17, miR- 20a, miR-20b, miR-93, miR-106a, miR-886-3p	down-regulated in ALK(+) ALCL versus ALK(−) ALCL: miR-155	
ALK(+) cell lines (SU-DHL-1, KiJK, KARPAS 299 and SR-78) and ALK(−) ALCL cell line (Mac-1)	CD3+ T-cells from healthy donors	Next generation sequencing	up-regulated in ALK(+) versus ALK(−) ALCL: miR-106a, miR-1246, miR-135b, miR-135b*, miR-139-5p, miR-145, miR-145*, miR-181a-2*, miR-182, miR-183, miR-183*, miR-1910, miR-203, miR-20b, miR-20b*, miR-223, miR-25*, miR-3182, miR-320d, miR-335*, miR-339-3p, miR-363, miR-3938, miR-4326, miR-501-3p, miR-548i, miR-548n, miR-548t, miR-574-3p, miR-574-5p, miR-582-5p, miR-629*, miR-874, miR-9, miR-9*, miR-92a-2*, miR-96	down-regulated in ALK(+) versus ALK(−) ALCL: miR-98, miR-942, miR-937, miR-766, miR-625*, miR-625, miR-542-3p, miR-513c, miR-513a-5p, miR-505, miR-503, miR-497, miR-450b-5p, miR-450a, miR-424*, miR-365, miR-34a, miR-342-5p, miR-342-3p, miR-33b*, miR-33b, miR-3200-3p, miR-3194, miR-301b, miR-26a, miR-2355-5p, miR-221, miR-2110, miR-210, miR-21*, miR-205, miR-199a-5p, miR-196b, miR-196a, miR-195, miR-194, miR-193b, miR-192, miR-181a, miR-155, miR-149, miR-146a, miR-1301, miR-1271	[35]
			up-regulated in ALK(+) ALCL versus T-cells: miR-98, miR-96, miR-937, miR-92a-2*, miR-9*, miR-9, miR-629*, miR-574-5p, miR-574-3p, miR-549, miR-548t, miR-548n, miR-548i, miR-542-3p, miR-503, miR-501-3p, miR-450b-5p, miR-450a, miR-4326, miR-424*, miR-3938, miR-365, miR-363, miR-34a, miR-33b*, miR-3200-3p, miR-3182, miR-301b, miR-25*, miR-210, miR-21*, miR-20b*, miR-20b, miR-203, miR-196a, miR-193b, miR-1910, miR-183*, miR-183, miR-182, miR-149, miR-145*, miR-145, miR-139-5p, miR-135b*, miR-135b, miR-1246, miR-106a	down-regulated in ALK(+) ALCL versus T-cells: miR-1271, miR-1301, miR-146a, miR-155, miR-181a, miR-181a-2*, miR-192, miR-194, miR-195, miR-196b, miR-199a-5p, miR-205, miR-2110, miR-221, miR-223, miR-2355-5p, miR-26a, miR-3194, miR-320d, miR-335*, miR-339-3p, miR-33b, miR-342-3p, miR-342-5p, miR-497, miR-505, miR-513a-5p, miR-513c, miR-582-5p, miR-625, miR-625*, miR-766, miR-874, miR-942	

**Table 2:** List of microRNA (miRNA) whose expression is dysregulation in ALK(+) ALCL.

microRNA	Expression	microRNA targets and their function	Ref.
miR-101	Downregulated	mTOR: Central regulator of cellular metabolism, growth and survival. Mcl-1: Anti-apoptotic protein	[33]
miR-135b	Overexpressed	FOXO1: Transcriptional activator, regulates cell cycle inhibitors. GATA3: Transcriptional activator, required for the Th2 differentiation process following immune and inflammatory responses. STAT6: Transcriptional activator; involved in differentiation of Th2 cells.	[30]
miR-150	Downregulated STAT3-dependent Epigenetic silencing by DNMT1	MYB: Transcriptional activator, controls the proliferation and differentiation of hematopoietic cells	[37]
miR-155	Downregulated	C/EBPβ: Transcriptional activator, regulates the expression of genes involved in the immune and inflammatory responses SOCS1: Regulator of cytokine signal transduction; involved in the negative regulation of cytokines that signal through the JAK/STAT3 pathway	[37]
miR-16	Downregulated	VEGF: Growth factor, promotes cell proliferation and migration, apoptosis and the permeabilization of blood vessels	[38]
miR-17~92	Overexpressed, STAT3-dependent	BIM: Pro-apoptotic protein	[29,34]
miR-181a	Downregulated, C/EBPβ-dependent	Unknown: Thought to be involved in T-cell differentiation and modulating strength of TCR signalling	[35]
miR-203	Overexpressed, C/EBPβ-dependent	SOCS3: Negative regulator of cytokine signal transduction	[35]
miR-21	Downregulated	DNMT1: DNA methyltransferase	[39]
miR-219	Downregulated	ICOS: Enhances T-cell responses	[40]
miR-26a	Downregulated	iNOS: Produces the messenger molecule NO (nitric oxide)	[41]
miR-29a	Downregulated STAT3-dependent Epigenetic silencing by DNMT1 and DNMT-3B	Mcl-1: Anti-apoptotic protein	[42]
miR-96	Down-regulated	ALK: Tyrosine kinase	[22]

**Table 3:** List of coding genes silenced by DNA methylation in ALK(+) ALCL.

Gene	Cellular Function	Promoter Methylation Silencing	Models	Ref.
*P16 ^INK4a^*	Cell cycle	*NA*	ALCL cell lines	[56]
*TNF-α*	Proinflammatory cytokine	*NA*	ALCL cell lines	[64]
*NFATC1*	Pro-apoptotic	*NA*	ALCL cell lines	[55]
*BIM*	Pro-apoptotic	*NA*	ALCL cell lines and tumor biopsies	[57]
*SHP1*	Negative regulator of TCR signaling	STAT3- and DNMT1–dependent	ALCL cell lines	[58,60]
*STAT5A*	Signal transduction and activation of transcription	STAT3- and DNMT1-dependent	ALCL cell lines and tumor biopsies	[59]
*ILR2-γ*	T-cell differentiation	STAT3- and DNMT1-dependent	ALCL cell lines and tumor biopsies	[39]
*ZAP70*	Component of TCR	STAT3-dependent	ALCL cell lines	[61]
*CD3*	Component of TCR	STAT3-dependent	ALCL cell lines	[58]
*SLP76*	Component of TCR	STAT3-dependent	ALCL cell lines	[58]
*CD28*	TCR co-stimulatory protein	STAT3-dependent	ALCL cell lines and tumor biopsies	[62]
*CTLA-4*	TCR co-stimulatory protein	STAT3-dependent	ALCL cell lines and tumor biopsies	[39]
